# Hydrogen sensing enhancement of zinc oxide nanorods via voltage biasing

**DOI:** 10.1098/rsos.172372

**Published:** 2018-05-23

**Authors:** Thye Foo Choo, Nur Ubaidah Saidin, Kuan Ying Kok

**Affiliations:** Materials Technology Group, Industrial Technology Division, Malaysian Nuclear Agency, Bangi 43000 Kajang, Selangor, Malaysia

**Keywords:** ZnO, hydrogen sensor, chemical, bias voltage

## Abstract

The capability of zinc oxide (ZnO) as a hydrogen sensing element has been pushed to its limits. Different methods have been explored to extend its sensing capability. In this paper, we report a novel approach which significantly improves the hydrogen sensing capability of zinc oxide by applying a bias voltage to ZnO nanorods as the sensing elements. Zinc oxide in the form of aligned nanorods was first synthesized on an Au-coated Si(111) substrate using a facile method via the galvanic-assisted chemical process. The sensing performance of the zinc oxide nanorods was investigated in response to the applied biasing voltage. It was found that the sensitivity, response time and detection limit of the ZnO sensing elements were dramatically improved with increasing bias voltage. A 100% increment in sensing response was achieved for the detection of 2000 ppm hydrogen gas when the bias voltage was increased from −2 to −6 V with 70% reduction in response and recovery times. This remarkable sensing performance is attributed to the reaction of hydrogen with chemisorbed oxygen ions on the surface of the ZnO nanorods that served as the electron donors to increase the sensor conductance. Higher reverse bias voltages sweep the electrons faster across the electrodes. This shortened the response time and, at the same time, depleted the electrons in the sensor elements and weakens oxygen adsorption. The oxygen ions could then be readily removed by hydrogen, leading to a higher sensitivity of the sensors. This, therefore, envisages a way for high-speed hydrogen gas sensing with high detection sensitivities.

## 1. Introduction

As one of the II–VI compounds, zinc oxide (ZnO) has a wide energy band gap of 3.37 eV, and a large exciton binding energy of 60 meV. It has a hexagonal wurtzite-type structure and possesses a high degree of flexibility in growth geometries. ZnO in the form of one-dimensional (1D) nanostructure, in particular, exhibits electrical and optical anisotropy as well as high electron mobility. These unique characteristics have made ZnO suitable for many device applications including gas sensor, solar cell and photodetector. Nanostructured ZnO can be fabricated by various methods such as chemical vapour deposition [1], electrochemical deposition [2], chemical [3,4], electrospinning [5] and hydrothermal [6], with the microstructures and properties of the materials affected by the growth mechanisms involved in the corresponding methods.

Applications of zinc oxide in hydrogen gas sensing have widely been studied over the past two decades, starting with the use of ZnO thin films with heating elements [7–10] incorporated to 1D nanostructures based on ZnO nanorods/nanowires as the sensing elements [11–21]. It has been reported that gas sensors based on 1D nanostructures are more superior in gas detection than thin film-based gas sensors because of their large surface-to-volume ratios of the former and also their dimensions are comparable to those of the gas analytes being analysed. Consequently, the binding of an analyte to the surface of a 1D nanostructure would result in the depletion or accumulation of carriers across the thickness of the nanostructure instead of only a particular surface region of a bulk or a thin film being affected. This gives rise to larger change in the resistance/conductance and higher sensitivity in gas detection when 1D nanostructures are used as the sensing materials.

Research efforts on gas sensing have been focusing on the improvement of sensing performance by improving their sensing characteristics such as the sensitivities, response and recovery times as well as detection limits of the sensors. For example, methods such as surface modification [11,16], catalyst doping [18], Schottky junction incorporation [19,20] and piezotronic integration [22,23] have been employed to achieve good sensing performance. However, the effects of operating bias voltages of sensors on the hydrogen sensing behaviours of the materials have rarely been investigated. For example, multimeters were commonly used in determining the changes in resistance during sensing events [12,13,16,24–27]. However, the multimeter is unable to perform both sourcing and measuring tasks simultaneously. Thus, the scope of evaluation of the sensors’ performance was constrained by the instrumental limitations. A source measure unit (SMU) is a more suitable instrument for sensing performance characteristics when compared with a multimeter. Although SMUs have been used in some of the works, only constant bias voltages were applied throughout the whole duration of sensing measurements [11,21,28,29], and many did not clearly state the sensors' operating bias voltages in their reports [15,17–20].

The main aim of this work is to investigate the effect of bias voltage on the hydrogen gas sensing performance of ZnO nanorods. One-dimensional nanostructures based on vertically aligned ZnO nanorods were fabricated on Au-coated Si substrates by a facile one-pot galvanic-assisted technique as proposed by Zheng *et al*. [24]. Field emission scanning electron microscope (FESEM) and X-ray diffractometry (XRD) were used to characterize the microstructure, orientation and phase formation of ZnO. Hydrogen sensing behaviour of the material was characterized by exposing the material to various concentrations of hydrogen gas with applied sensor's bias voltages ranging from −2 to −6 V.

## 2. Materials and methodology

Au-coated silicon substrate was cleaned using standard procedures by first sonicating in acetone for 15 min followed by isopropyl alcohol for 15 min before rinsing with deionized water (DI) and blown dry with air. The edges of the substrate were then wrapped with Al foils so that the difference in the reduction potentials between the two materials provided the driving force for the formation of the ZnO nanostructures by galvanic displacement reaction. An aqueous solution, containing 25 mM zinc nitrate hexahydrate (Zn(NO_3_)_2_·6H_2_O, 98%) and 25 mM hexamethylenetetramine (C_6_H_12_N_4_), was used as the electrolyte. The solution was maintained at 75°C in a water bath on a hotplate. The substrate was placed with growth surface facing downward in the electrolyte and the growth time for ZnO was 4 h. The ZnO-coated silicon substrate was then rinsed with DI water and blown dry with air. The ZnO nanorod coating was characterized using an X-ray diffractometer (PANalytical X'Pert Pro MPD) with Cu K*α* radiation. The morphology of the nanorods was examined using a FESEM (Carl Zeiss GeminiSEM 500).

**Figure 1.**
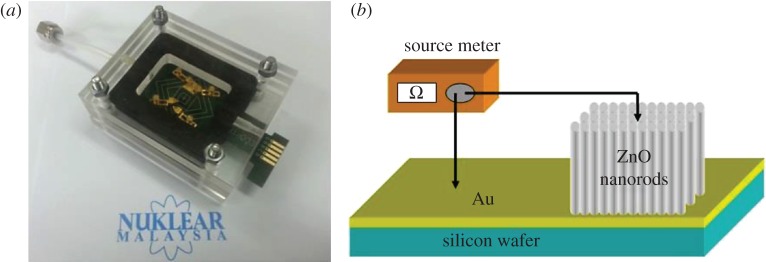
(*a*) A sensor chip with mechanical contact electrode mounted on a PCB inside the custom-made gas chamber. (*b*) Schematics of the experimental set-up for hydrogen sensing measurements.

For gas sensing measurements, the substrate with ZnO nanostructures was mounted on a pre-fabricated printed circuit board (PCB) with the Au-coated Si substrate as the mechanical contact electrode (figure 1*a*). Figure 1*b* shows the schematic diagram of the fabricated sensor chip. Gas sensing measurements were performed with constant gas flow across the sensor chip in a sealed custom-made acrylic glass chamber of volume 12 cm^3^ (figure 1). The hydrogen gas was diluted with different proportions of dry air (purity: 99.998%) and regulated by a mass flow controller at a flow rate 200 SCCM (standard cubic centimetres per minute). Gas sensing data were acquired via a customized Labview program interfaced with a Keithley Source Measure Unit 2602A. A range of bias voltages from −2 to −6 V was applied to the sensors. All the experiments were carried out with the sensor chip first exposed to air to obtain the baseline resistance, followed by exposures to the desired concentrations of hydrogen gas before the air was flushed back to complete a cycle. Time interval for each H_2_ flow and air purging event was set to 300 s. The sensitivity (*R*) of the ZnO sensor towards H_2_ gas was defined as the percentage of relative resistance change ([(*R*_g_ – *R*_0_)/*R*_0_]), using the following equation:

2.1 $$\text{sensitivity},\;R(\%)=\left[\frac{\left(R_{g}-R_{0}\right)}{R_{0}}\right]\times 100,$$

where *R*_0_ and *R*_g_ are the sensor resistances in the absence and presence of H_2_ gas, respectively.

The response time is defined as the time required for reaching 90% of the total change in the electric resistance at a given H_2_ concentration, while the recovery time is defined as the time required for reaching 10% of the original baseline value after the removal of H_2_.

## 3. Results and discussion

### 3.1. Characterization of the ZnO nanorods

**Figure 2.**
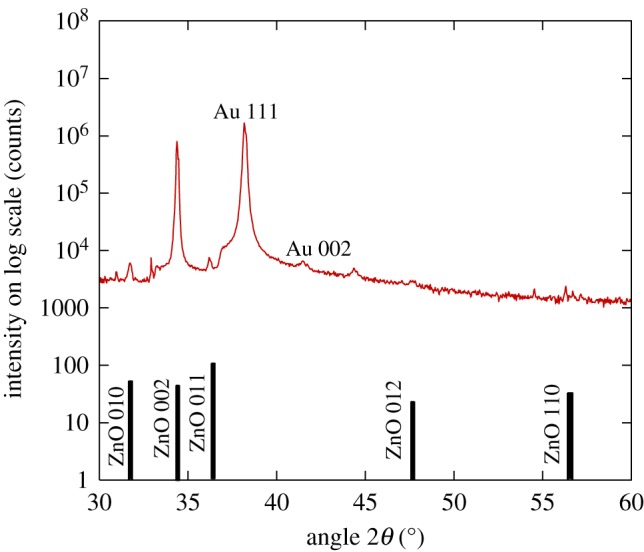
X-ray diffractogram of ZnO grown on Al-wrapped Au-coated Si substrate. The positions of the ZnO diffraction peaks as obtained from the standard reference pattern (ICSD card no. 980009346) are indicated by the stick pattern.

**Figure 3.**
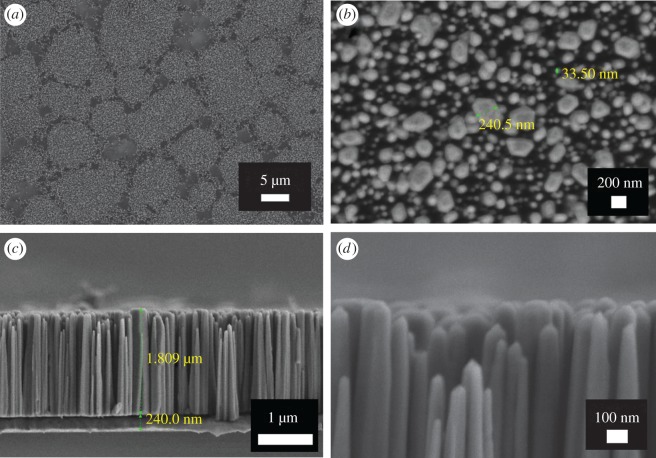
(*a*) Top-view FESEM image of the ZnO sample, (*b*) high-magnification image of (*a*), (*c*) cross-sectional view of the sample and (*d*) high-magnification cross-sectional view of ZnO nanorods.

X-ray diffractions performed on the sample show that ZnO nanorods were grown as pure crystalline phase of wurtzite structure with a preferred growth along (002) (figure 2). Figure 3 shows the FESEM micrographs of ZnO nanorods grown on Au-coated Si substrate. Top view FESEM image of the sample in figure 3*a* reveals that the Au-coated Si substrate was not fully covered by ZnO nanorods. The nanorods formed numerous clusters of different sizes. On close examination, the nanorods in figure 3*b* exhibit irregular shapes, diameters ranging from 30 to 240 nm with an average of 103 nm. The cross-sectional view micrograph in figure 3*c* reveals that the ZnO nanorods are perpendicularly oriented to the substrate with an average length of 1.8 µm. The thickness of the Au coating is approximately 240 nm. As shown in figure 3*d*, the majority of the smaller ZnO nanorods tend to be cylindrical with sharper tips compared to the larger ones.

In a chemical process, the formation of ZnO starts with the reduction reaction of dissolved oxygen (equation (3.1)) followed by the formation of Zn(OH)_2_ (equation (3.2)) which subsequently converts to ZnO via dehydration (equation (3.3)). In this work, the difference in electronegativity between Al and the Au-coated Si, provided the driving force for the electrons from Al to move to the substrate so that the reduction reaction of dissolved oxygen occurred more efficiently. When the concentration of Zn^2+^ and OH^−^ exceeded supersaturation, ZnO nuclei formed at the interface between the substrate and the electrolyte solution. The substrate orientation in the electrolyte, on the other hand, played an important role in activating the anisotropy growth of ZnO nuclei along the [001] direction to form orthogonally grown ZnO nanorods. The formation of ZnO nanorods can be described by the following reactions:

3.1 $${\text{O}}_{2}+2{\text{H}}_{2}\text{O}+4{\text{e}}^{-}\to \text{4O}{\text{H}}^{-},$$

3.2 $$\text{Z}{\text{n}}^{2+}+\text{2O}{\text{H}}^{-}\to \text{Zn(OH}{\text{)}}_{2}$$

3.3 $$\;\mathrm{and}\;\text{Zn(OH}{\text{)}}_{2}\to \text{ZnO}+{\text{H}}_{2}\text{O}.$$

**Figure 4.**
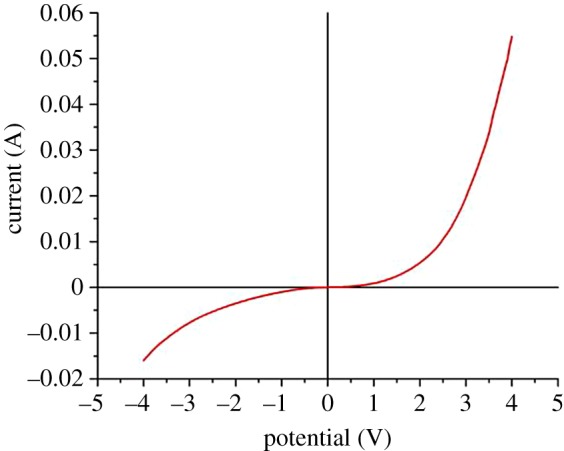
Typical *I*–*V* characteristic curve of the ZnO nanorod arrays.

Figure 4 shows the typical *I*–*V* characteristics of the ZnO nanorod arrays on Au-coated silicon substrate grown at room temperature. The *I*–*V* plot shows the rectifying Schottky behaviour due to the presence of ZnO/Au heterojunction. Reverse bias voltages of −2, −4 and −6 V were used to characterize the hydrogen sensing behaviour of ZnO as forward bias gave lower sensing characteristics when exposed to H_2_ gas. Yu *et al*. [25,30] also reported that faster sensing response was obtained in reverse bias compared to forward bias operation.

### 3.2. Hydrogen gas sensing

**Figure 5.**
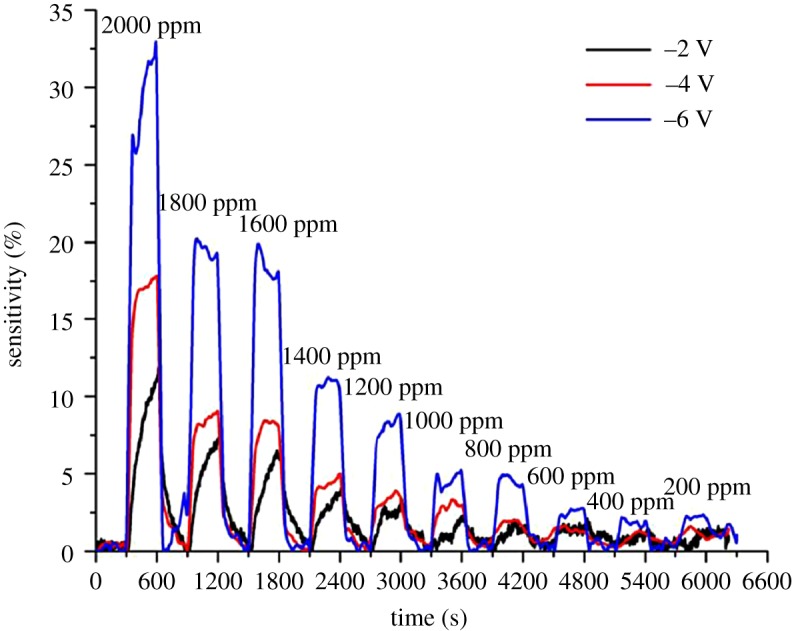
Sensitivity plotted against time when the ZnO sensor was exposed to hydrogen at different concentrations at room temperature at different reverse bias voltages.

**Figure 6.**
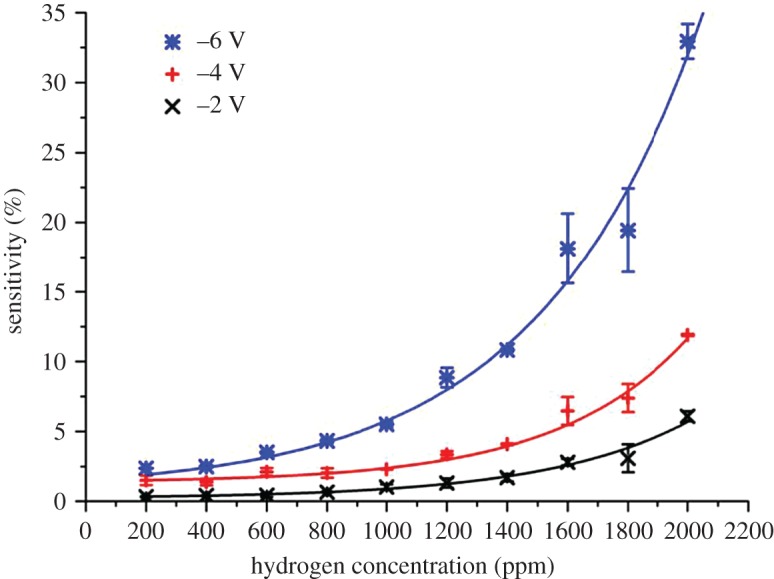
Comparison of the sensitivity (%) as a function of H_2_ concentration at room temperature at different reverse bias voltages.

Figure 5 compares the sensitivity of ZnO nanorods towards different concentrations of H_2_ at room temperature at bias voltages of −2, −4 and −6 V. The sensitivity to hydrogen increases significantly with increasing reverse bias voltage, while the response and recovery times decrease significantly. Resistance change is still prominently visible at hydrogen concentration as low as 200 ppm. However, the sensitivity observed at the concentrations of 1800 and 1600 ppm appeared to be almost similar. The sensitivity observed at 1000 and 800 ppm as well as sensitivity observed between 600 and 200 ppm (figure 6) are also not significantly differentiated. This could be attributed to the incomplete gas mixing deficiency during the measurement.

**Figure 7.**
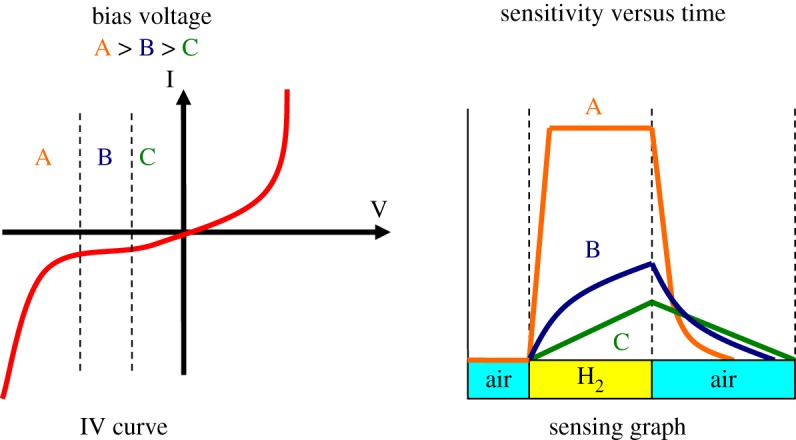
Hydrogen sensing behaviours at different bias voltages. Higher sensitivity and shorter response time and recovery time were obtained for ZnO sensor operated at larger bias voltage.

**Figure 8.**
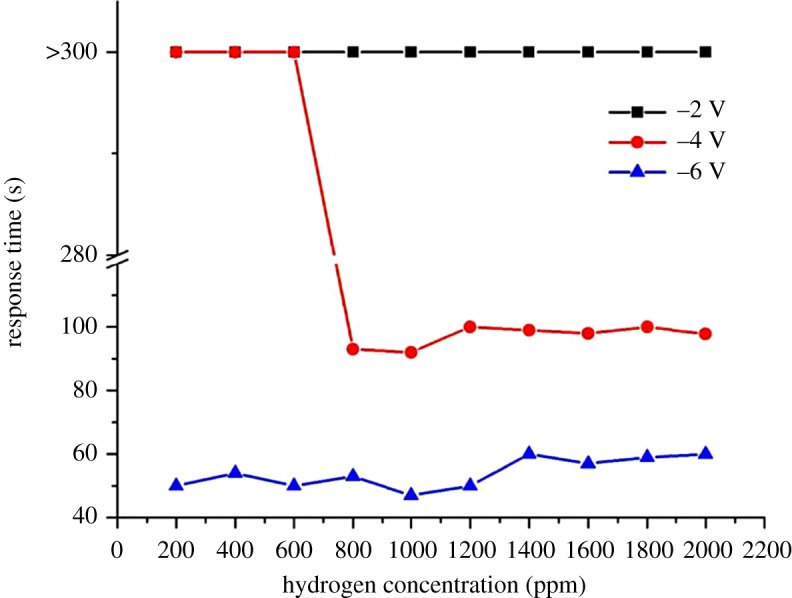
Comparison of the response time (s) as a function of H_2_ concentration at room temperature at different reverse bias voltages.

**Figure 9.**
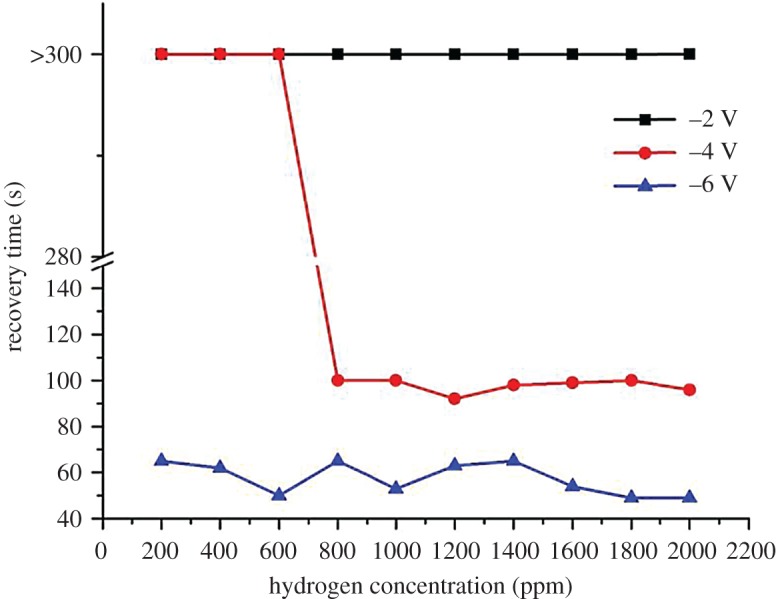
Comparison of the recovery time (s) as a function of H_2_ concentration at room temperature at different reverse bias voltages.

Figure 7 illustrates the differences in hydrogen sensing behaviours of ZnO at different bias voltages. A 100% increment in the sensor's sensitivity was recorded at 2000 ppm of hydrogen gas when the bias voltage increased from −2 to −6 V. Reduction in the response and recovery times of more than 70% was also recorded (figures 8 and 9). Dependency of the sensing characteristics on bias voltage provides a promising route to achieving high-speed and optimum sensing performance. This could be attributed to the effects of the interactions between hydrogen, chemisorbed oxygen ions and electro-migration in the sensor materials. ZnO is a well-known n-type semiconductor with its electrons contributed by oxygen vacancies and Zn interstitials [31]. When exposed to the atmospheric environment, the electrons from the ZnO conduction band ionized the atmospheric oxygen to produce negative oxygen ions at the surface of the ZnO nanorods:

3.4 $${\text{O}}_{2}\;\text{(gas) + }{\text{e}}^{-}\to {{\text{O}}_{2}}^{-}\;(\text{ads}).$$

This led to a decrease in electron concentration resulting in an increase in the surface resistance. A depletion layer was then formed between the immobile oxygen ions and the zinc ions. Upon exposure to hydrogen gas, H_2_ reacted with the negatively charged oxygen ions to produce H_2_O molecules by consuming chemisorbed oxygen from the nanorods' surface. This interaction increased the conductivity of ZnO nanorods by releasing the chemisorbed O_2_ electrons back to the ZnO conduction band.

3.5 $${{\text{O}}_{2}}^{-}\;(\text{ads})+2{\text{H}}_{2}\;(\text{gas})\to 2{\text{H}}_{2}\text{O}\;(\text{gas})+{\text{e}}^{-}.$$

**Figure 10.**
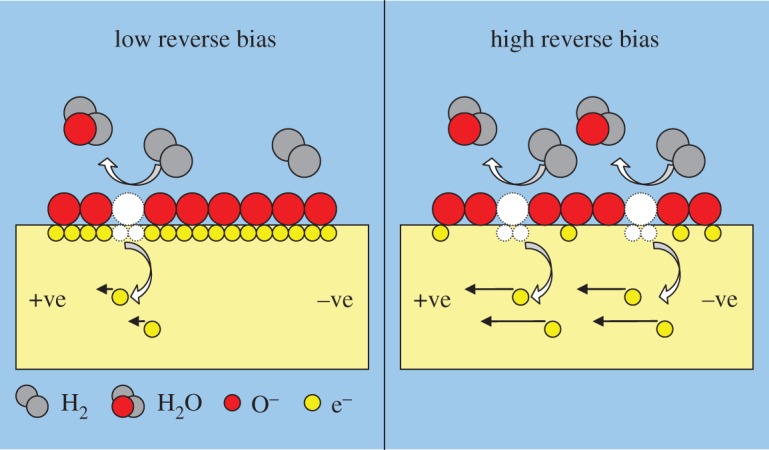
Schematics of the sensing mechanisms of the nanorods at low and high reverse bias voltages.

At higher reverse bias, the released electrons moved at a faster rate due to the greater driving force across the electrodes and thus, decreased the response times. At the same time, reverse bias also depleted the electrons in the nanorods and weakened oxygen adsorption resulting in oxygen ions being easily removed by H_2_ leading to an improvement in sensor performance. Recovery time of a gas sensor is determined by the rate at which atmospheric oxygen recombines with the electrons on the ZnO surface. Owing to the relatively low number of electrons available at high reverse bias, the recovery time is shorter compared to that at low reverse bias. Figure 10 shows the schematics of the proposed sensing mechanisms involved.

A comparison of sensitivity, response time and recovery time for hydrogen gas sensor in this work (table 1) with the reported results from previous studies [11,17,26,27,29,32,33] is shown in table 2. The sensor reported in this work has the shortest response and recovery times, while the sensitivity of the sensor is also higher when compared to others that used ZnO nanorods as the sensing materials except for Hassan *et al.* [33]. Despite that, their ZnO sensor showed poorer recovery and response times. A high sensitivity sensor is suitable for quantitative measurement of gas concentrations, whereas a fast response sensor, as demonstrated by the sensor characteristics in this work, will be suitable for fast sensing and detection of gas leakage.

**Table 1.** Sensitivity, response time and recovery time of the sensor measured at different reverse bias voltages of −2, −4 and −6 V for different H_2_ concentrations.

	–2 V			–4 V			–6 V		
H_2_ (ppm)	Δ*R*/*R*_o_ (%)	response time (s)	recovery time (s)	Δ*R*/*R*_o_ (%)	response time (s)	recovery time (s)	Δ*R*/*R*_o_ (%)	response time (s)	recovery time (s)
2000	11.91	>300	>300	17.83	98	96	32.94	60	49
1800	7.39	>300	>300	9.05	100	100	19.44	59	49
1600	6.47	>300	>300	8.23	98	99	18.13	57	54
1400	4.09	>300	>300	5.00	99	98	10.82	60	65
1200	3.35	>300	>300	3.85	100	92	8.85	50	63
1000	2.30	>300	>300	3.03	92	100	5.25	47	53
800	2.03	>300	>300	1.99	93	100	4.32	53	65
600	2.11	>300	>300	1.32	>300	>300	2.78	50	50
400	1.38	>300	>300	1.24	>300	>300	1.93	54	62
200	1.50	>300	>300	1.02	>300	>300	2.37	50	65

**Table 2.** Comparison of the sensitivity, response and recovery times between the present sensor and other existing sensors operated at room temperature.

reference	materials	operating voltage (V)	response time (s)	recovery time (s)	sensitivity (%)/ H_2_ (ppm)
this work	oriented ZnO nanorods	6.0	60	49	33/2000
Wang *et al*. [11]	ZnO nanorods	0.5	>300	<20	5/500
Hassan *et al.* [17]	ZnO nanorods	—	400	187	0.4/20 000
Kadhim *et al.* [29]	spin coat SnO_2_	0.2	192	95	2570/1000
Fields *et al.* [32]	nanobelts SnO_2_	0.1	220	—	60/20 000
Xiang *et al.* [26]	Pd/TiO_2_ nanotubes	—	120	90	8/10 000
Wang *et al.* [27]	Nb_2_O_5_ nanowires	—	100	470	100/2000
Hassan *et al.* [33]	ZnO nanorods	0.1	176	116	500/1000

## 4. Conclusion

In this paper, we have investigated the room temperature hydrogen gas sensing behaviour of ZnO nanorods grown on Au-coated Si(111) substrate by the galvanic-assisted chemical process. It was found that the hydrogen sensing behaviour and performance of the sensor depended strongly on the applied bias voltage. The sensitivity, response time and recovery time could be improved many folds of magnitude by using large bias voltages. This paves the way for the fabrication of hydrogen gas sensors with good detection sensitivities and low detection limits for high-speed sensing in real time.

## Data accessibility

Our data are deposited at http://dx.doi.org/10.5061/dryad.hr804 [34].
